# Unilateral lower limb atrophy associated with glomus tumors: a case report

**DOI:** 10.1186/s13256-020-02616-1

**Published:** 2021-01-13

**Authors:** Yuji Akechi, Shiroh Miura, Masayuki Ochi, Moe Enoki, Takuya Matsuda, Riko Kitazawa, Taketsugu Fujibuchi, Hirofumi Ochi, Michiya Igase, Yasumasa Ohyagi

**Affiliations:** 1 Department of Neurology and Geriatric Medicine, Ehime University Graduate School of Medicine, Shitsukawa, Toon, Ehime 791-0295 Japan; 2 Department of Radiology, Ehime University Graduate School of Medicine, Toon, Ehime Japan; 3 Department of Molecular Pathology, Ehime University Graduate School of Medicine, Toon, Ehime Japan; 4 Department of Bone and Joint Surgery, Ehime University Graduate School of Medicine, Toon, Ehime Japan

**Keywords:** Unilateral, Lower limb atrophy, Pain, Amyotrophy, Glomus tumor, Monoplegia

## Abstract

**Background:**

Glomus tumors are soft tissue neoplasms comprised of glomus cells, vasculature, and smooth muscle cells, which occur commonly in a single subungual area of the digits, and their main clinical features include severe paroxysmal pain, localized tenderness, and cold hypersensitivity.

**Case presentation:**

A 47-year-old Japanese man had suffered from chronic progressive paroxysmal shooting pain in his right leg since childhood. He avoided putting weight on his right foot whenever he walked. The frequency of paroxysmal pain and the number of tender points both gradually increased with age, and his right leg gradually atrophied. Magnetic resonance imaging of the lower extremity demonstrated multiple gadolinium-enhanced nodules that corresponded with his tender points. Excisional biopsy relieved his pain and provided a histopathological diagnosis of glomus tumors.

**Conclusion:**

This case suggests that small glomus tumors located in deep tissue may cause disuse atrophy because of their long delay before diagnosis. Clinicians should consider the potential for glomus tumors when patients exhibit unilateral lower limb muscular atrophy with pain.

## Background

Glomus tumors are soft tissue neoplasms comprised of glomus cells, vasculature, and smooth muscle cells. These tumors commonly occur in a single subungual area of the digits, and their main clinical features include severe paroxysmal pain, localized tenderness, and cold hypersensitivity [1, 2]. Of patients with single extradigital glomus tumors, 41% were observed in the lower extremities [2]. Further, 10% of patients with glomus tumors had multiple tumors, more than 50% of whom had a family history, and in most cases an extremity was involved [3]. Herein, we present a sporadic patient with multiple glomus tumors showing unilateral lower limb muscular atrophy.

## Case presentation

A 47-year-old Japanese man was admitted to our hospital presenting with progressive excruciating pain and atrophy in his right leg. He had no family history of pain or amyotrophy. Since childhood he had noticed a sharp tingling sensation from his right ankle to the lateral thigh when he tapped his right lateral malleolus. Walking for long periods often induced languor and pain in the rear side of his right lower extremity. During his teenage years he also experienced an occasional localized sharp shooting pain in his right lower extremity when walking. Both the frequency of paroxysmal severe pain and the number of trigger points gradually increased with age. Although he consulted with various medical institutions, the cause of pain was undiagnosed. He started to take pain relievers at 41 years old. His right lower limb gradually atrophied during his clinical course, while there was no change in his skin color, swelling, or feeling of heat in the affected limb.

**Fig. 1.**
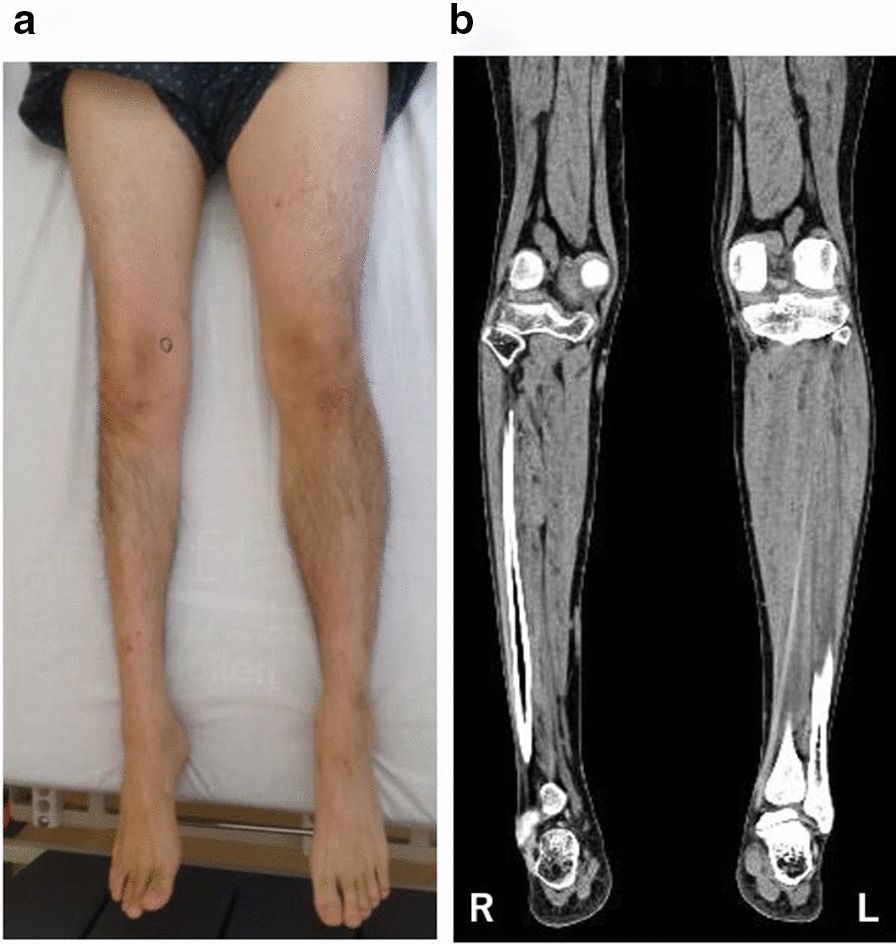
Muscular atrophy of the patient’s right leg. **a** Photograph. **b** Computed tomography (CT)

His daily medicines at hospitalization were tramadol hydrochloride (75 mg), acetaminophen (650 mg), pregabalin (150 mg), duloxetine hydrochloride (40 mg), eperisone hydrochloride (150 mg), and clonazepam (0.5 mg). He also took pilsicainide hydrochloride hydrate (50 mg) and carteolol hydrochloride (5 mg) during episodes of pathological tachycardia caused by severe leg pain. At neurological examination his pupils were dilated and were slow to light stimulation. The convergence reflex was intact. Ptosis was not observed. Examination of other cranial nerves identified no abnormalities. His cerebellar function was normal. Diffuse muscle atrophy in the right lower extremity (Fig. 1a) and contracture of the right Achilles tendon were observed. However, the reflex of the right Achilles tendon was not examined because of unbearable pain. Other deep tendon reflexes were normal except for a slightly brisk bilateral patellar tendon reflex. Pathological reflexes were not found. His superficial and deep sensations were both normal.

The patient had four points of highly localized tenderness and sensitivity to cold or pressure. The first was the right lateral aspect of his lower leg (7 cm proximal to the lateral malleolus), which appeared during junior high school. The second was the distal portion of his right lateral Achilles tendon, which appeared during high school. The third was the right dorsal aspect of his lower leg (11 cm distal to the right head of the peroneal bone), which appeared at 23 years old. The fourth was his right popliteal fossa, which appeared at 42 years old. He experienced continuous mild tingling at and around these tender points. During walking he maintained an ankle dorsiflex posture of his right limb, and landed with the outside of his sole.

**Fig. 2.**
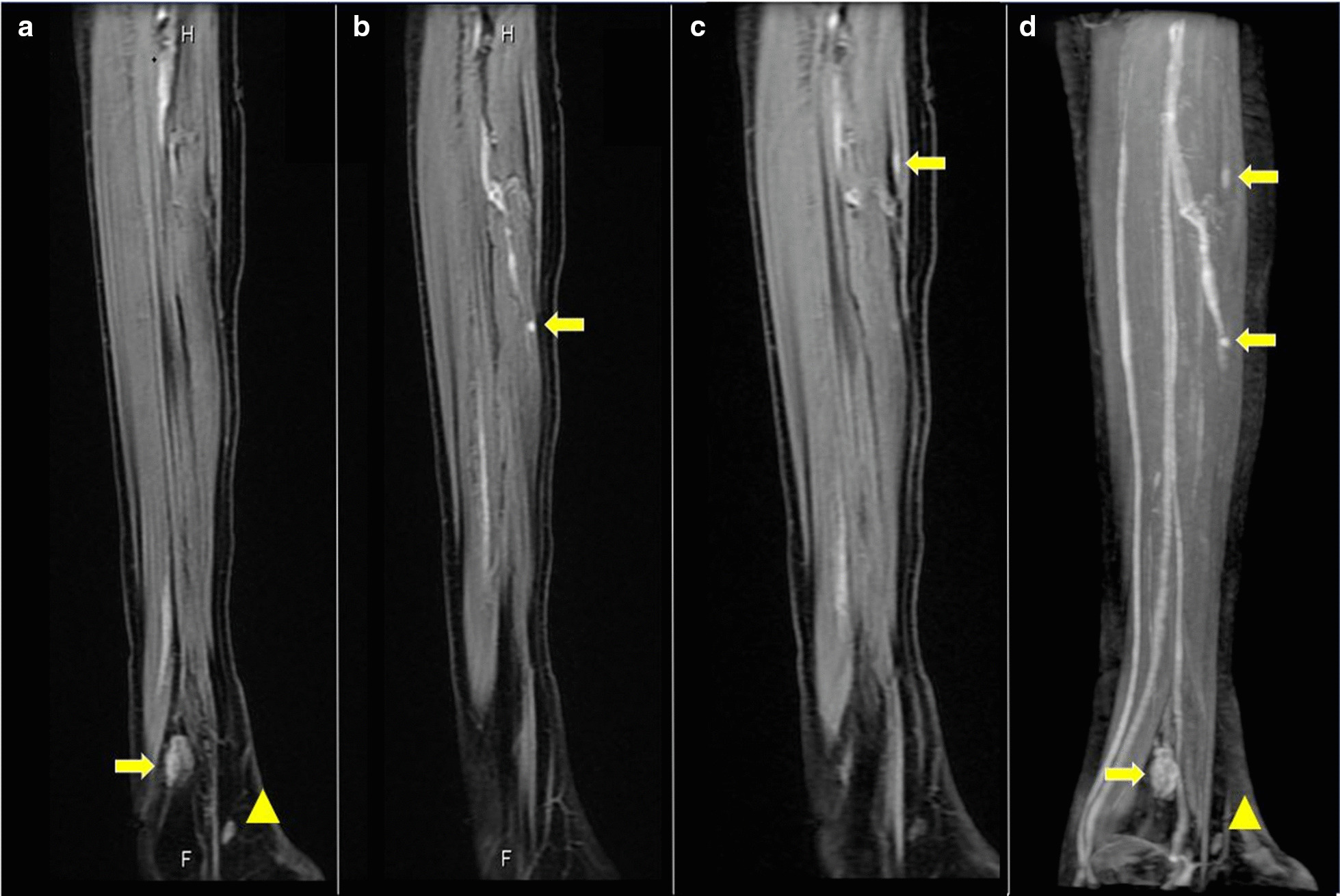
Sagittal gadolinium (Gd)-enhanced fat-suppressed T1-weighted magnetic resonance imaging (MRI) of the patient’s right lower leg. **a** Two enhanced nodules were found near the ankle joint. One was a flat nodule in the deep tissue between the peroneal bone and the shin bone above the ankle joint (arrow). The other was a small nodule in the dorsiflexion side of the foot joint (arrowhead). **b**, **c** Small enhanced nodules in the gastrocnemius muscle (arrows). **d** Maximum intensity projection images of (**a**−**c**). The images are water-only images obtained using the modified two-point Dixon method

**Fig. 3.**
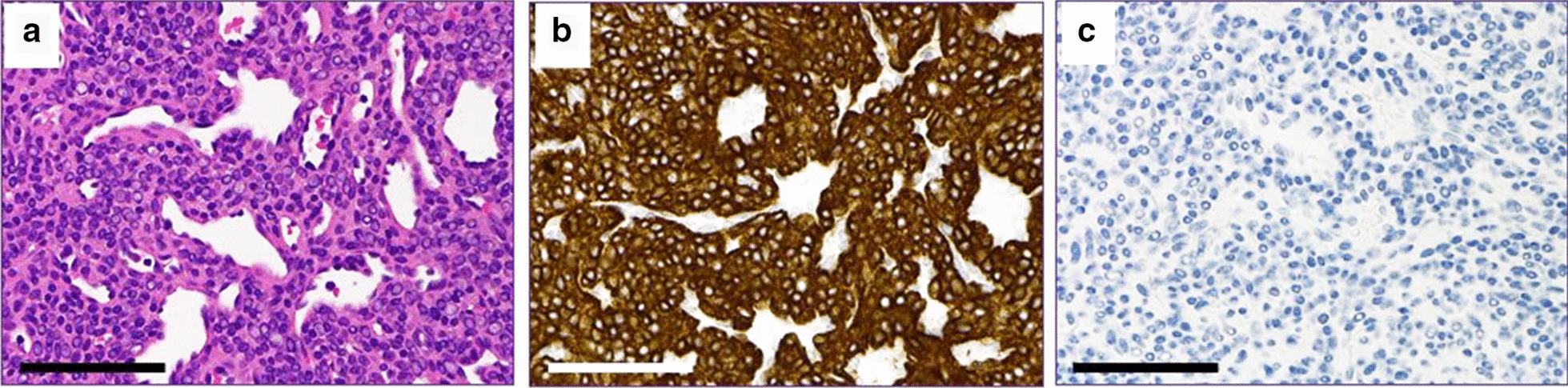
Histochemical and immunohistochemical staining of the tumor. **a** Hematoxylin and eosin staining. Comparatively uniform cells with a round nucleus surrounding the vascular space were observed. **b** Immunohistochemical staining using the anti-smooth muscle actin antibody using diaminobenzidine (DAB). The tumor cells were positive. **c** Immunohistochemical staining with anti-desmin antibody using DAB. The tumor cells were negative. The blue color reflects the hematoxylin counter stain. Scale bars, 100 µm

His serum creatine kinase was 59 U/L (male normal range, 59−248 U/L). Nerve conduction studies (including F-wave) were normal in the bilateral tibial and peroneal nerves, while the compound muscle action potential amplitudes were lower in the right tibial and peroneal nerves compared with the left. Sensory nerve action potential amplitude and sensory conduction velocity were normal in both sural nerves. Needle electromyography showed no abnormalities in the right tibialis anterior, rectus femoris, or left rectus femoris muscles. Computed tomography of his lower extremity demonstrated right leg atrophy (Fig. 1b). Magnetic resonance imaging of his right lower leg showed multiple gadolinium-enhanced nodules (Fig. 2). The patient underwent excisional biopsy for two lesions (Fig. 2a). On pathological examination, the tumor cells were positive for smooth muscle actin and negative for desmin (Fig. 3), indicating glomus tumors [1]. After surgical excision he could walk with his right sole touching the ground, and his bilateral mydriasis had fully recovered at discharge.

## Discussion

The three main features of our case were: (i) a gradually progressive, paroxysmal, and localized shooting pain caused by glomus tumors, which was improved by surgical resection, (ii) unilateral lower limb muscular atrophy, and (iii) bilateral mydriasis. The first feature was compatible with the typical clinical course of glomus tumors [2, 3, 5–7]. Several reports have also described unilateral lower limb muscular atrophy associated with glomus tumors [4–7], although there are limited neurological findings. The unilateral lower limb muscular atrophy associated with glomus tumors may relate to peripheral nerve damage [4]. Alternatively, the involved extremity may have a vasomotor disturbance due to long-standing pain, as observed in complex regional pain syndrome [8]. Further, avoiding use of the involved extremity may cause atrophy of disuse. In our case, needle electromyography for checking the possibility of myopathy and motor neuron disease revealed no pathological neurogenic or myogenic changes despite progressive limb atrophy associated with chronic (approximately 40 years) right lower limb pain. Thus, disuse atrophy may have caused the unilateral lower limb muscular atrophy in our patient. To our knowledge, this case has the longest reported disease duration until diagnosis. This is likely related to the small tumor size and the deep tissue location, and may explain his unilateral whole leg atrophy. We propose two possibilities for the bilateral mydriasis observed at admission. First, the patient was in the prodromal stage of serotonin syndrome because of his chronic tramadol hydrochloride and duloxetine treatment. Second, the patient may have experienced sympathicotonia caused by chronic severe pain.

## Conclusion

Clinicians should consider the possibility of multiple cryptogenic glomus tumors when patients complain of unilateral lower limb muscular atrophy accompanied with paroxysmal lightening pain.

## Data Availability

The datasets used in the current study is available from the corresponding author on reasonable request.
